# Characterization of the Sodium Multi-Vitamin Transporter in the Mosquito *Anopheles stephensi* and Its Capacity to Mobilize Pantothenate and Biotin

**DOI:** 10.3390/biom15010059

**Published:** 2025-01-03

**Authors:** Jun Isoe, Brendan F. Riske, Megan E. Dobson, Hannah L. Kaylor, Jessica C. Brady, Yared Debebe, Laura M. Saavedra, Shirley Luckhart, Michael A. Riehle

**Affiliations:** 1 Department of Entomology, University of Arizona, Tucson, AZ 85721, USA; jisoe@ag.arizona.edu (J.I.); brendanriske1@gmail.com (B.F.R.); lsaavedra31@arizona.edu (L.M.S.); 2 Department of Biological Sciences, University of Idaho, Moscow, ID 83844, USA; dobs2343@vandals.uidaho.edu (M.E.D.); brad3269@vandals.uidaho.edu (J.C.B.); 3 Department of Entomology, Plant Pathology and Nematology, University of Idaho, Moscow, ID 83844, USA; kayl8810@vandals.uidaho.edu (H.L.K.); ydesta@uidaho.edu (Y.D.); sluckhart@uidaho.edu (S.L.)

**Keywords:** SMVT, transport, *Anopheles stephensi*, vitamin B5, vitamin B7, pantothenate, biotin, malaria, mosquito, survival, reproduction

## Abstract

Pantothenate (Pan), or vitamin B5, is essential for the synthesis of co-enzyme A (CoA), acetyl-CoA, and numerous downstream physiological processes. We previously demonstrated that Pan is not only essential for mosquito survival, but also for the development of malaria parasites within the mosquito, suggesting that targeting Pan and CoA biosynthesis may be a novel approach for malaria control. However, little is known about how Pan is acquired and mobilized within the mosquito. In this work, we examined Pan levels in the important human malaria vector *Anopheles stephensi*, including the abundance of Pan during immature development and adulthood. We also assessed the distribution of Pan in various adult tissues and examined the impact of provisioning Pan to the mosquito via a sugar or blood meal on mosquito survival and reproduction. Furthermore, we examined how Pan was mobilized in the mosquito via a putative Pan transporter, the *A. stephensi* sodium multi-vitamin transporter. We demonstrated that this transporter is capable of mobilizing both Pan and biotin (vitamin B7) in a dose dependent manner. We also assessed the distribution of *A. stephensi* sodium multi-vitamin transporter in the mosquito and its capacity to transport vitamins. This work establishes the basic physiology of Pan uptake and mobilization in the mosquito, providing essential information for Pan based malaria control strategies.

## 1. Introduction

Malaria continues to kill over 600,000 people annually and this number is likely to increase as current control strategies fail and the ecological distribution of highly competent mosquito vectors continues to expand [1,2]. Current malaria control strategies largely rely on insecticides, including insecticide treated bednets and indoor residual spraying, along with a limited repertoire of drugs and vaccines. Unfortunately, these strategies are becoming less effective as insecticide and drug resistance increase [1]. At the same time, important malaria vectors such as *Anopheles stephensi* are making dramatic expansions into malaria endemic areas in Africa, greatly increasing the risk of transmission [2]. Thus, it is essential that we identify novel mosquito-targeted approaches for malaria control. Malaria parasites must acquire all nutrients required for their development and multiplication from the vertebrate and mosquito hosts. This provides an opportunity to starve malaria parasites of essential nutrients in either host to block their development. One of these essential nutrients is pantothenate (Pan) or vitamin B5 [3]. We previously demonstrated that increasing the conversion of Pan to co-enzyme A (CoA) in *A. stephensi* using small molecule activators (pantazines) of the rate limiting enzyme pantothenate kinase (PanK) significantly reduced available Pan in adult mosquitoes [4,5]. This Pan reduction in turn led to reduced malaria parasite infection prevalence and intensity in *A. stephensi* [4]. This manipulation did not affect key factors of mosquito fitness including reproduction, lifespan, and metabolism^4^, since CoA was still available to the mosquito [5]. However, while increased PanK activity in mammalian tissues has been shown to induce uptake of exogenous Pan [6], little is known about how Pan is transported into mosquito cells to fuel increased conversion to CoA.

Pan is synthesized by plants and some microbes, leading to an excess of available Pan in the human diet [7]. Pan-containing plant nectar (pers. comm.) and vertebrate blood [4] consumed by adult mosquitoes are perhaps a necessity based on our data, which suggest that adult female *A. stephensi* gut microbiota do not produce significant Pan [4]. Regardless of source, however, there is little to nothing known about the abundance and distribution of Pan during the immature and adult mosquito stages or its availability to mosquito-stage malaria parasites. In this context, how Pan is absorbed by the midgut and mobilized to various tissues in the mosquito remains a black box. In humans, Pan is transported via a sodium multivitamin transporter (SMVT). Human SMVT is a membrane bound protein consisting of thirteen transmembrane domains that are widely distributed in most tissues, particularly in absorptive tissues such as the kidneys, liver, colon, intestine, and placenta [8]. It is a member of the larger solute carrier 5 (SLC5) gene family, whose twelve members transport amino acids, vitamins, sugars, and smaller organic molecules across cell membranes [9]. The human SMVT transports two vitamins that cannot be synthesized de novo, Pan and biotin (vitamin B7), in addition to α-lipoic acid and iodide [10]. While SMVT is the primary transporter for Pan and biotin, it is a secondary transporter for iodide. In addition, lipoic acid can be synthesized in mitochondria [10], making transmembrane transport dispensable. Both Pan and biotin compete for SMVT, with an excess of one vitamin inhibiting the transport of the other. In humans, biotin can also be transported by a monocarboxylate transporter (MCT) [11] and high biotin levels can suppress SMVT mRNA expression to reduce biotin uptake [10].

The only ortholog of human SMVT described from arthropods is the *Drosophila melanogaster* SMVT (CG42235, DmelSMVT). Dutta et al. examined DmelSMVT knockdown effects in the fly intestine and found that RNAi treatment led to “shrunken” midguts [12]. Further, when DmelSMVT was specifically knocked down in intestinal stem cells (ISCs), a loss of midgut ISCs was observed within seven days [12]. In 2022, Neophytou and Pitsouli demonstrated that these effects could be rescued by expressing human SMVT in the fly and that biotin dysregulation led to changes in the gut microbiome [13]. However, these studies focused only on DmelSMVT as a biotin transporter, and we could find no studies on the role DmelSMVT in Pan transport.

In this study, we identified and characterized a single putative *A. stephensi* SMVT ortholog (AsteSMVT; ASTEI07648) and, based on our results and the highly conserved functions of SMVTs noted above, we propose that this is the primary Pan transporter in *A. stephensi*. In addition, we assessed Pan levels and uptake during a variety of mosquito life stages and physiological events to establish baseline levels of Pan in the mosquito. Combined, this study offers valuable insights into a poorly studied facet of mosquito physiology, which may open new avenues towards disrupting malaria parasite development and transmission.

## 2. Materials and Methods

### 2.1. Mosquito Stocks and Maintenance

*A. stephensi* (Indian strain) was maintained at 27 °C and 80% humidity with light/dark cycling. Adult mosquitoes were provided with either 10% sucrose solution via cotton pads ad libitum (replaced daily) or water-soaked cotton pads and sugar cubes. For colony maintenance, adult female *A. stephensi* were provisioned with whole bovine blood with citrate anticoagulant (Hemostat, Dixon, CA, USA) via glass membrane feeders or were allowed to feed on CD-1 mice (Envigo, St. Louis, MO, USA) sedated with ketamine (50 mg/kg) and xylazine (5 mg/kg). Mouse protocols were performed at the University of Idaho and approved by the Animal Care and Use Committee and were in accordance with federal regulatory guidelines and standards (University of Idaho IACUC-2023-8 protocol, approved 27 February 2023). For non-infectious experimental blood feeding, mosquitoes were provided with whole bovine blood via membrane feeders or type O+ washed human red blood cells (RBCs) and human serum (1:1 vol:vol) via glass membrane feeders. Human RBCs and serum were purchased from a commercial vendor (Grifols Bio Supplies, Inc., Los Angeles, CA, USA). In preparation for mosquito feeding, contents of blood bags were transferred to sterile 50 mL conical tubes using a Baxter 4C2243 Fenwal Plasma Transfer unit (VWR Scientific, Radnor, PA, USA). Blood aliquots were diluted 1:1 (vol:vol) with RPMI 1640 medium (ThermoFisher Scientific, Carlsbad, CA, USA) and mixed by inverting several times. Tubes were then centrifuged at 4 °C at 1000× *g* for 10 min, followed by aspiration of supernatant and white protein layer above the RBCs. Washing with RPMI 1640 medium was completed three times prior to mixing with serum for mosquito feeding. RBCs were used within two weeks of washing. Human serum was heat-inactivated at 56 °C for 30 min, then aliquoted and stored at −80 °C prior to use. Adult female mosquitoes used in these studies were 3–7 d old unless otherwise noted.

### 2.2. Assessing Pan and Biotin Levels in Mosquitoes

Pan assays were conducted using the standard assay for Pan in foodstuffs from AOAC International [14], which we previously optimized for mosquito tissues [5]. Mosquito whole bodies (adults, larvae, and pupae), midguts, fat bodies/abdominal body walls, head/brain, and ovaries were dissected from wild-type or dsRNA treated mosquitoes. Tissues were homogenized in 200 µL of homogenization buffer (100 mM acetate buffer (pH 4.6) papain (20 mM), α-amylase (8.5 mM), 2% chloroform), incubated overnight at 37 °C, autoclaved, and stored at 4 °C until Pan quantification. *Lactobacillus plantarum* (ATCC 8014), which requires Pan for growth, was cultured in MRS broth at 37 °C for 24 h, pelleted at 4000 g for 10 min, and resuspended in 1 mL sterile saline (0.9% (*w*/*v*) NaCl). The resuspended bacteria (2 µL) were added to 2 mL of Difco assay medium deficient in Pan (BD, East Rutherford, NJ, USA). Calcium D (+) Pan was added to create a standard curve (0, 5, 10, 20, 40, 80, and 160 ng/mL medium). Mosquito tissue extracts with unknown Pan levels were added to *L. plantarum* seeded media and allowed to grow at 37 °C for 20 h. Turbidity was measured at OD550 and compared against the standard curve to quantify Pan. Assays were replicated at least four times with separate cohorts of *A. stephensi*. Pan levels were analyzed by ANOVA and Tukey’s test or unpaired Student’s *t*-test. Biotin assays were conducted as described above for Pan, since biotin is also essential for *L. plantarum* growth. To determine biotin levels, we grew *L. plantarum* in Difco assay medium deficient in biotin (BD, East Rutherford, NJ, USA). All other assay steps were conducted as described for the Pan assay. Wing measurements were conducted as previously described [15]. For dry weight measurements, pupae and adults (*n* = 20 per sample) were transferred to pre-weighed 1.5 mL microcentrifuge tubes. Samples were centrifuged at 1000 rpm for 1 min and excess water removed from the pupae samples. Tubes with their lids open were placed in an incubator (37 °C; Eppendorf, Hauppauge, NY, USA) for 5 d to dry the samples and measured on a 0.1 mg balance (Sartorius, Bohemia, NY, USA).

### 2.3. Pan Provisioning and Excretion Assays

To assess the capacity of adult female mosquitoes to acquire Pan during a blood meal, we placed mosquitoes in a modified 50 mL Falcon tube with the conical end removed and replaced with screen mesh and a removable 35 mm petri dish in place of the screw cap. We then provided mosquitoes with no blood meal, an unsupplemented blood meal, or a blood meal supplemented with 10, 100, or 1000 ng/µL Pan. The petri dish was replaced after 1 h once a majority of blood meal diuresis was complete. Subsequently, we replaced the plastic petri dish every 24 h throughout the mosquito reproductive cycle (up to 96 h) and imaged each petri dish. Fecal material containing excreted Pan was washed from the petri dish by pipetting up and down three times with 100 mM acetate buffer (pH 4.6) without papain and α-amylase. Pan levels in the feces were quantified using the above Pan assay (2.2). Each mosquito cage contained ten fully engorged females, and three unique biological cohorts were assayed. One-way ANOVA and Tukey’s post hoc tests were used to identify significant differences.

### 2.4. Determining AsteSMVT Expression in Mosquitoes

*AsteSMVT* expression was examined in the midgut, fat body, and ovaries of *A. stephensi* females. Tissues were dissected in 1X PBS from mosquitoes prior to blood feeding and at 2, 6, 12, 24, 36, 48, 72, and 96 h after blood feeding. Total RNA was extracted using TRIzol reagent, and 250 ng of DNaseI-treated total RNAs were reverse transcribed using an oligo-(dT) primer and reverse transcriptase. The cDNA was then used as a template for qPCR assays using gene-specific primers (Table S1). Briefly, qPCR was performed with Maxima SYBR Green/ROX qPCR Master Mix with a final primer concentration of 200 nM using a CFX Connect qPCR machine (Biorad, Hercules, CA, USA). The following PCR conditions were used: 95 °C for 5 min followed by 40 cycles of 95 °C for 10 s and 56 °C for 30 s. Ribosomal protein S7 transcript levels were used as an internal control for normalization of mRNA yields in all samples. Three biological replicates were performed with unique mosquito cohorts.

### 2.5. Knockdown of AsteSMVT Using RNAi

RNAi was used to knockdown *AsteSMVT* to expand our understanding of AsteSMVT-dependent Pan and biotin uptake and transport. DNA templates for double-stranded RNA (dsRNA) synthesis were generated for both *AsteSMVT* and the firefly luciferase (*Fluc*) control, using gene-specific forward and reverse *AsteSMVT* primers (Table S1) and whole-body female *A. stephensi* cDNA as a template. A T7 RNA Pol promoter sequence TAATACGACTCACTATAGGGAGA was added to the 5′ end of each primer (Eurofins Genomics, Louisville, KY, USA) prior to PCR with Taq 2X Master Mix (NEB, Ipswich, MA, USA). The resulting PCR templates were ligated into the pGEM-T easy cloning vector (Promega, Madison, WI, USA) and positive clones sequenced for accuracy. The dsRNA was transcribed in vitro using the *AsteSMVT* and *Fluc* templates, NTP nucleotides, and T7 RNA polymerase from HiScribe™ T7 Quick High Yield RNA Synthesis Kit (NEB). Cold-anesthetized female mosquitoes were microinjected twice with 2.0 μg of dsRNA at 4 h and 3 d after adult emergence using a Nanoject II microinjector (Drummond Scientific Company, Broomall, PA, USA). Mosquitoes were allowed to recover in a humid environment and were maintained on 10% sucrose during the experiments.

### 2.6. Mosquito Reproduction Studies

*Provisioning of Pan* via *blood:* Control and treatment groups of 100–120 female mosquitoes were transferred 3 d after adult emergence into 2 L polypropylene containers topped with mesh screening. These mosquitoes were maintained for 14 d on 10% sucrose-soaked cotton balls. On day 14, mosquitoes were offered a blood meal (washed human RBCs and heat-inactivated serum 1:1 vol:vol) via an artificial feeder. Treated mosquitoes received a blood meal supplemented with 100 ng/µL Pan in filtered, distilled water, while controls received a blood meal without Pan. Sucrose-soaked cotton balls were removed 30 min prior to bloodfeeding, and mosquitoes were allowed to bloodfeed for 30 min, after which non-fed mosquitoes were removed and quantified. At 48 h post-feeding, up to 75 bloodfed mosquitoes were transferred to individual 50 mL centrifuge tubes containing 2 mL water and allowed to oviposit for 72 h, after which they were returned to the master cage. The presence of eggs in each tube was recorded, and tubes with eggs were photographed and eggs counted. The proportions of female mosquitoes laying eggs were analyzed using Fisher’s exact test between groups. The numbers of eggs per female were analyzed with Welch’s *t*-test. Four biological replicates were completed with separate cohorts of *A. stephensi*.

*Provisioning of Pan* via *sugar meal:* For provisioning Pan via sugar meal, the above protocol was modified as follows. Up to 11 d post-eclosion, adult mosquitoes were maintained on 10% sucrose-soaked cotton balls. In other experiments, three days prior to blood feeding, 11 d old mosquitoes were provided 100 ng/µL Pan in water via saturated cotton balls along with a sugar cube as a source of carbohydrates. Control mosquitoes were provided water-soaked only cotton balls along with a sugar cube. Cotton balls were changed twice daily, and mosquitoes were primed with Pan for 3 d prior to the blood meal. After Pan priming, 14 d old females were blood fed and egg production quantified as above.

### 2.7. Effect of Pan Provisioned in Water or Blood on Male and Female A. stephensi Lifespan

The effect of Pan supplementation on the lifespan of male and female *A. stephensi* was determined. The effects of Pan supplementation in water were evaluated through provisioning Pan in water-soaked cotton balls, with sugar cubes provided as a carbohydrate source. Three-day old male and female *A. stephensi* were provided with 0.05, 0.5, 5, and 50 g/L Pan in filtered, distilled water-soaked cotton balls that were replaced twice daily for three consecutive days. A control group was provided with water only. Three biological replicates were conducted using unique cohorts of mosquitoes for a total of 200 mosquitoes (replicate 1 = 40 mosquitoes; replicates 2 and 3 = 80 mosquitoes each). Females were also provided with washed, type O+ human RBCs supplemented with 1:1 heat-inactivated serum each week and allowed to oviposit. The impact of Pan provisioning via blood feeding was evaluated by providing *A. stephensi* with a weekly blood meal (washed human RBCs and heat-inactivated serum 1:1 vol:vol) via an artificial feeder. Treated mosquitoes received a blood meal supplemented with 10, 100, or 1000 ng/µL Pan in filtered, distilled water, while controls received a blood meal without Pan. The first blood meal was offered to 3 d old female *A. stephensi*, and non-blood fed mosquitoes were removed (first blood meal only). One day after each weekly blood meal, a petri dish lined with moistened filter paper was provided for oviposition. This was removed after three days. Four biological replicates were conducted using separate cohorts of mosquitoes for a total of 1443 mosquitoes (each replicate ≈ 400 mosquitoes). For both studies, dead mosquitoes were counted and removed daily until the last mosquito perished in each group. Data were analyzed using JMP Pro version 17 (SAS Institute Inc., Cary, NC, USA) and GraphPad Prism version 10.3.1 (GraphPad Software, Boston, MA, USA). Kaplan–Meier survival analysis was used to test the variation in survival distribution of mosquitoes in each cohort. Log-rank test was used to compare each treatment group with the control.

## 3. Results

### 3.1. Abundance of Pan During Mosquito Development

**Figure 1:**
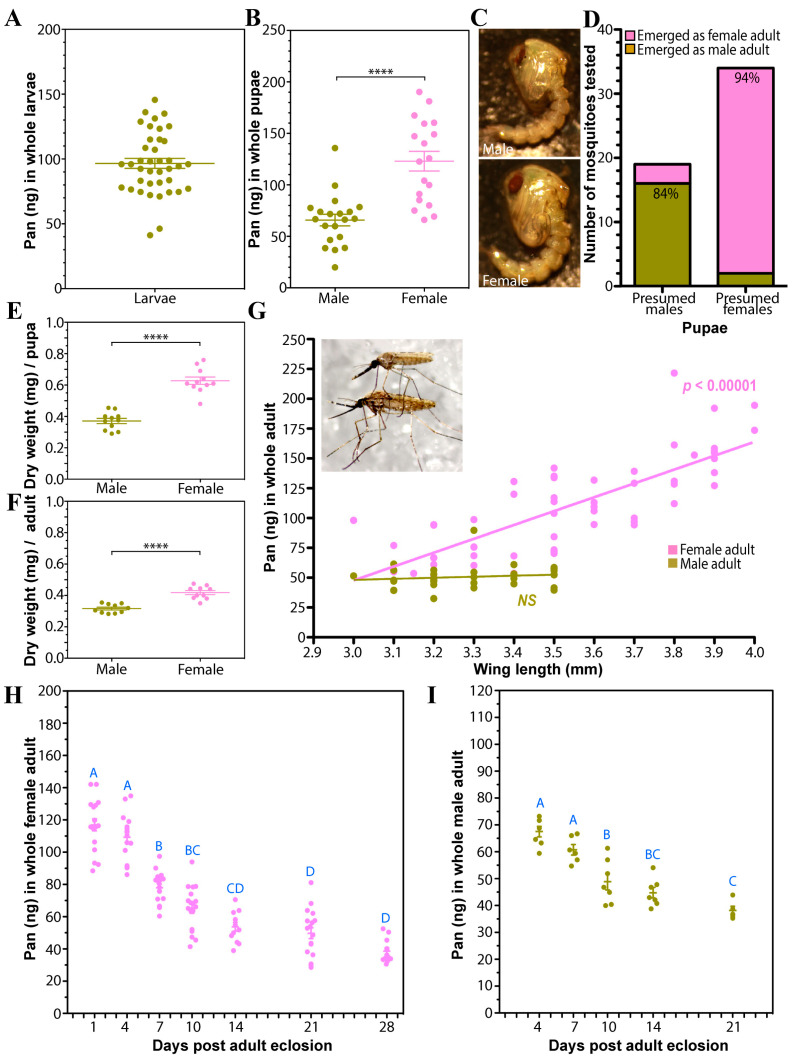
Pan levels in developmental stages of *A. stephensi*. (**A**). Larval Pan levels. Pan levels were measured in whole late 4th instar larvae. Larvae were thoroughly washed in distilled water prior to homogenization. N = 39. (**B**). Whole body Pan levels in pupae were measured from individuals that pupated within the last 24 h. Pupae were visually inspected and grouped into predicted males or females based on their size. **** = *p* < 0.0001. (**C**). Representative images of male (Top) and female (Bottom) pupae are shown. (**D**). A subset of pupae was randomly selected and allowed to eclose to determine their sexes. 84% of the putative male pupae emerged as male adults (16/19), while 94% of the putative female pupae emerged as female adults (32/34). Data were analyzed by unpaired Student’s *t*-test to assess whether female pupae had more Pan than male pupae (*p* < 0.0001). (**E**). Total dry weight of pupae after dehydration. Each dot represents a pool of 20 pupae. (**F**). Total dry weight measurement of male and female adults. Each dot represents a pool of 20 adult mosquitoes 1 d after eclosion. (**G**). Pan levels and wing length of female mosquitoes were significantly correlated, with larger female mosquitoes having higher Pan content. Wing length and Pan levels were measured individually for female and male mosquitoes. All mosquitoes had eclosed within 24 h. NS = not significant (**H**). Pan levels in aging female mosquitoes. Females were collected 1, 4, 7, 10, 14, 21, and 28 d after adult emergence. They were provided with 10% sucrose throughout the experiments. Each dot represents five pooled whole mosquitoes. Different letters indicate statistically significant differences (one-way ANOVA, N = 12–18). Pan levels steadily decreased as female adult mosquitoes aged. (**I**). Pan levels in aging male mosquitoes. Males were collected 4, 7, 10, 14, and 21 d after adult emergence. They were provided with 10% sucrose throughout the experiments. Each dot represents five pooled whole mosquitoes and bars represent the mean and standard error. Different letters indicate statistically significant differences (one-way ANOVA and Tukey’s post hoc, N = 12–18). As with females, Pan levels in males steadily decreased as they aged.

To establish baseline Pan levels throughout the mosquito development cycle, we determined Pan levels in larval, pupal, and adult mosquitoes. In *A. stephensi* 4th instar larvae, Pan levels varied considerably, with an average 96.9 ng per whole larva and a range of 40–140 ng/larva (Figure 1A; *n* = 39). This range of Pan is likely due to body size variability between smaller male and larger female larvae, which cannot be separated by sex at this stage. Indeed, in pupae, which can be sexed with reasonable confidence (94% female and 84% male prediction accuracy), we observed significantly more Pan in female pupae (123.0 ng) than male pupae (65.9 ng; Figure 1B,C). This 86% increase in average Pan levels in female pupae is largely in line with their ~70% larger body mass as assessed by dry weight (Figure 1E). Similarly, adult females possess significantly more Pan (115 ng) at 24 h post-eclosion than males (50 ng). As with pupae, adult female mosquitoes are larger (~30%; Figure 1F) than males, although this difference in mass does not entirely account for females having over double the average amount of Pan. The amount of Pan in female mosquitoes increased linearly with body size as determined using female wing length (Figure 1G). Interestingly, this positive correlation between body size and Pan levels was not observed in males. Finally, we assessed Pan levels in 10% sucrose-fed adult female and male mosquitoes as they aged. Due to a lack of Pan in the adult diet, Pan stores were gradually depleted in 10% sucrose-fed females reaching the lowest level 14 d post-eclosion, after which Pan levels did not change significantly (Figure 1H). In male mosquitoes, we also observed a significant decrease in whole-body Pan levels as the mosquitoes aged (Figure 1I).

### 3.2. Distribution of Pan in Adult Female Mosquitoes Prior to and During Reproductive Cycles

**Figure 2:**
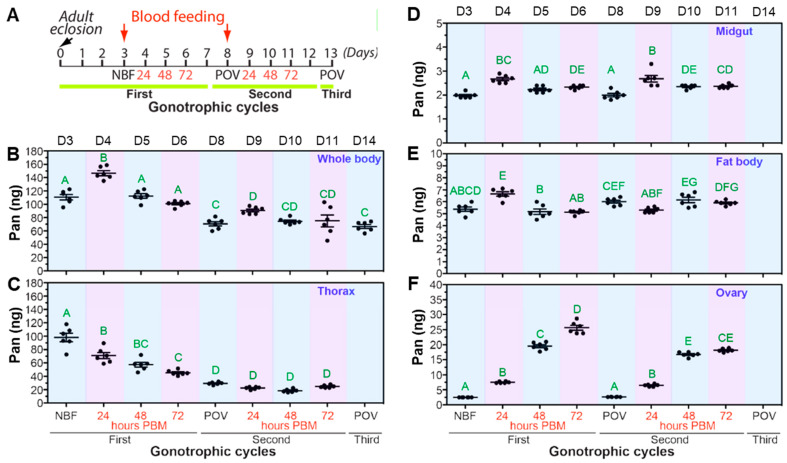
Pan levels in distinct tissues from *A. stephensi* females during the first two reproductive cycles. (**A**). Schematic representation of blood feeding protocol and tissue sample collection timepoints. Tissue samples were collected from the whole body (**B**), thorax (minus legs and wings; (**C**)), midgut (**D**), fat body/abdominal body wall (**E**), and ovaries (**F**) from non-blood fed (NBF) mosquitoes, at 24, 48, and 72 h post-blood meal (PBM), and 2 d post-oviposition (POV) over two gonotrophic cycles. Gravid females were allowed to lay eggs for 2 d at 72 h PBM. Approximately 5% of gravid females did not lay eggs after 2 d, and these females were excluded from analysis. Each dot represents five pooled tissues except for midguts where ten tissues were pooled for Pan quantification. Bars represent the mean and standard error. The day post-eclosion for each experimental time point is listed at the top of the graph. Different letters indicate statistically significant differences (one-way ANOVA with post-hoc Tukey, N = 6 per time point and three unique biological cohorts).

To assess the impact of blood feeding and reproductive cycles on Pan levels in female *A. stephensi*, we determined Pan levels in whole mosquitoes and in distinct tissues, including ovaries, midguts, the fat body/abdominal body wall, and the thorax (with legs and wings removed). Tissues were collected prior to blood feeding (NBF) and at 24, 48, and 72 h post-blood meal (PBM) over the first two reproductive cycles (Figure 2A). In the whole body, we observed an increase in overall Pan levels at 24 h PBM that quickly returned to pre-blood meal levels by 48 h (Figure 2B). We observed a similar pattern during the second gonotrophic, but with lower levels of Pan overall. In the thorax, there was a dramatic decrease in Pan levels throughout the first gonotrophic cycle (Figure 2C), suggesting that Pan was being depleted from this tissue for egg provisioning. Levels of Pan in the midgut (Figure 2D) and fat body (Figure 2E) were generally low, with modest but significant differences during a gonotrophic cycle, but no differences between the first and second gonotrophic cycles. Finally, in the ovaries, we observed moderate levels of Pan in the ovary that significantly increased during each day of the reproductive cycle. During the first reproductive cycle a significant increase in Pan was observed at 24, 48, and 72 h PBM. Interestingly, during the second reproductive cycle, we did not observe a significant increase in Pan late in the reproductive cycle (48–72 h PBM; Figure 2F), suggesting that Pan stores may be insufficient in this cycle to fully provision the developing oocytes.

### 3.3. Impact of Pan Supplementation in Adult Female A. stephensi via Sugar Meal or Blood Feeding

**Figure 3:**
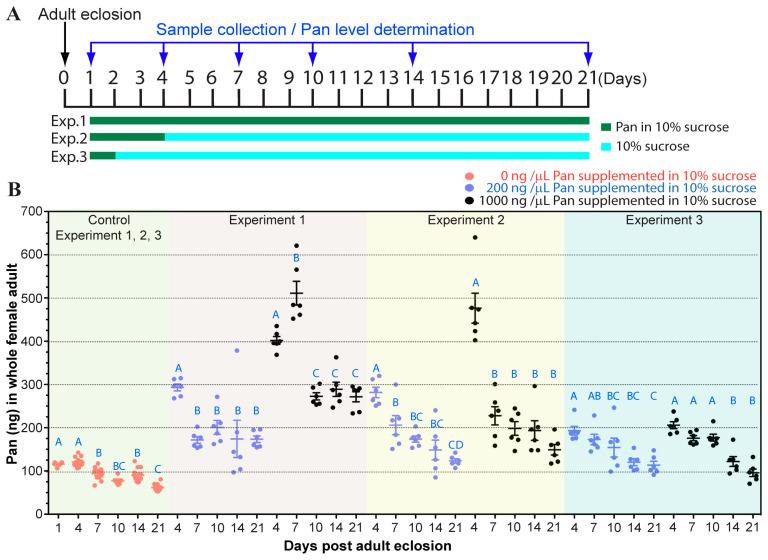
Pan levels in female *A. stephensi* supplemented with Pan in 10% sucrose. (**A**). A schematic showing three different Pan provisioning scenarios. In experiment 1, Pan in 10% sucrose was provided throughout the experiment. In experiment 2, Pan in 10% sucrose was provided only for the first 3 days. Thereafter, mosquitoes were only allowed to feed on 10% sucrose. In experiment 3, Pan in 10% sucrose was provided only on the first day. Thereafter, mosquitoes were only allowed to feed on 10% sucrose. Cotton balls soaked with 10% sucrose with or without Pan were changed daily to minimize microbial growth. (**B**). Female mosquitoes were supplemented with 0, 200, or 1000 ng of Pan/µL in 10% sucrose. Females were collected from 1, 4, 7, 10, 14, and 21 d post-adult eclosion and Pan levels were measured by microbiological assay using *Lactobacillus plantarum*. Each dot represents Pan levels from the five pooled whole mosquitoes and bars represent the mean and standard error. Different letters indicate statistically significant differences (one-way ANOVA and Tukey’s post-hoc test, N = 6–12).

In the above studies, female *A. stephensi* did not receive any Pan supplementation. Given that Pan is available in both nectar and blood, we sought to understand Pan uptake in females provisioned via a sugar meal or blood meal. We used three supplementation schemes to assess Pan uptake following provisioning in 10% sucrose (Figure 3A).

Immediately after adult eclosion, mosquitoes were given access to 10% sucrose supplemented with 0, 200 or 1000 ng/µL Pan for either throughout their adult life (Exp. 1) or for the first 72 h (Exp. 2) or 24 h (Exp. 3) after adult eclosion. For the 24 and 72 h treatments, mosquitoes were allowed access to 10% sucrose without Pan following the Pan treatment duration. Continuously provisioning adult females with 200 ng/µL Pan led to an increase to 300 ng Pan/mosquito at day 4, followed by consistent levels of ~180 ng/mosquito from days 7 to 21 (Figure 3B). A similar pattern was observed in mosquitoes continuously provisioned with 1000 ng/µL, although the peak occurred at 7 d and was higher (400–500 ng/mosquito), while Pan levels from days 10–21 remained near 300 ng/mosquito. For female mosquitoes provided with Pan via sugar feeding for the first three days of life only, we observed an initial increase in Pan levels at day 4 as with the continuous feeding. Pan levels subsequently dropped through days 7–21 yet remained higher than mosquitoes that received no Pan provisioning. Finally, mosquitoes provisioned with Pan for only the first 24 h after adult eclosion had elevated levels of Pan throughout their lifespan that were significantly higher than non-Pan provisioned controls until day 21.

**Figure 4:**
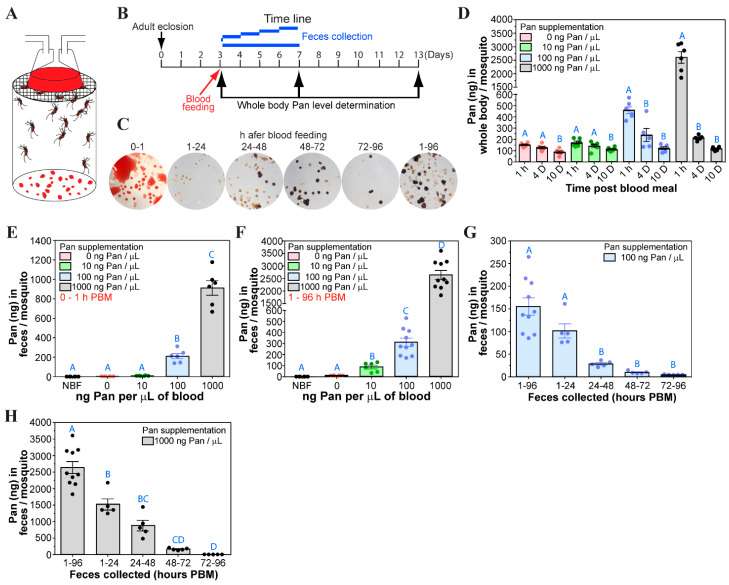
Pan levels in bodies and feces of *A. stephensi* supplemented with Pan in blood. (**A**). A schematic showing female mosquitoes taking a blood meal supplemented with Pan. Females were allowed to feed on warmed blood (40 °C) for 1 h through parafilm. (**B**). A schematic showing the timeline for Pan quantification in mosquito bodies and in feces following feeding. (**C**). Representative images of feces collected after blood feeding. Images of mosquito feces following feeding on unsupplemented blood. Feces from unsupplemented and supplemented mosquitoes were not visibly different. (**D**). Whole body Pan levels from mosquitoes fed on 0, 10, 100, or 1000 ng/µL Pan in blood. Pan quantification was performed on pools of 5 whole bodies at 1 h, 4 d, and 10 d PBM using the *Lactobacillus plantarum* assay. Feces were collected over the first hour (**E**) and from 1–96 h (**F**) PBM, dissolved in acetate buffer, and used to quantify excreted Pan. (**G**). Pan levels in feces from mosquitoes fed on Pan supplemented blood (100 ng/µL) and collected between 1 and 24 h, 24 and 48 h, 48 and 72 h, 72 and 96 h, and 1 and 96 h after blood feeding. (**H**). Pan levels in feces from mosquitoes fed on Pan supplemented blood (1000 ng/µL). Feces were collected between 1 and 24 h, 24 and 48 h, 48 and 72 h, 72 and 96 h, and 1 to 96 h PBM. Each dot represents individual samples and bars represent the mean and standard error. Different letters indicate statistically significant differences (one-way ANOVA and Tukey’s post hoc, N = 6–10).

We also examined Pan levels in female mosquitoes following a blood meal supplemented with Pan at multiple time points during the reproductive cycle up to 10 d PBM. We also examined the feces from the mosquitoes to assess the amount of Pan excreted. During the first hour PBM, *A. stephensi* excrete excess water, including lysed RBCs, to eliminate excess weight, producing bright red droplets (Figure 4A–C). Once feeding is complete, only a small amount of fecal excretion is observed (1–24 h PBM) as digestion proceeds. From 24–48 h PBM, we observed increased fecal material, including undigested blood, and this continues until the end of the reproductive cycle (48–72 h PBM), after which there is minimal excretion (72–96 h PBM). We did not detect any visual differences in fecal excretion between mosquitoes provided unsupplemented blood or blood supplemented with Pan. At 1 h PBM, high levels of Pan were observed in whole mosquitoes provisioned with 100 or 1000 ng/µL Pan (Figure 4D) relative to controls (no Pan) or blood supplemented with 10 ng/µL Pan, likely due to Pan retained in the midgut. By 4 d PBM, following digestion and excretion of the blood bolus, elevated Pan levels were still observed in mosquitoes that fed on blood with 100 or 1000 ng/µL Pan. However, by 10 d PBM there were no significant differences in whole body Pan levels among any of the treatments. Increased Pan levels at 1 h and 4 d PBM for mosquitoes supplemented with 100 and 1000 ng/µL Pan were not ten-fold higher than levels in mosquitoes treated with 10 ng/µL Pan, suggesting that considerable amounts of Pan were not retained by the mosquito.

To assess Pan excretion, we measured Pan levels in mosquito feces. During the first hour PBM, including feeding time, feces from control mosquitoes (no Pan) contained an average of 2.8 ng of Pan per mosquito (Figure 4E). In contrast, feces from mosquitoes provisioned with 10, 100, or 1000 ng/µL Pan excreted an average of 9.9, 210, and 911 ng Pan per mosquito within the first hour of feeding. Since a fully engorged *A. stephensi* female consumes an average of 3.7 µL of blood [16], this suggests that in the first hour, mosquitoes excrete 27%, 57%, or 25% of Pan provisioned at 10, 100, or 1000 ng/µL, respectively. Over the next 96 h, mosquitoes provisioned with 10, 100, or 1000 ng/µL Pan excreted an average of 44, 155, or 2639 ng per mosquito (Figure 4F). This excretion decreased over time as the total amount of Pan remaining in the mosquito decreased (Figure 4G,H). Taken together, dietary Pan is acquired by female *A. stephensi* in amounts likely based on endogenous levels that decline with age. Further, excretion appears to be the primary mechanism to manage Pan levels when intake exceeds amounts required for normal function. Based on this, we focused on characterizing the putative *A. stephensi* Pan transporter.

### 3.4. Identification of a Putative Sodium Multi-Vitamin Transporter in A. stephensi

**Figure 5:**
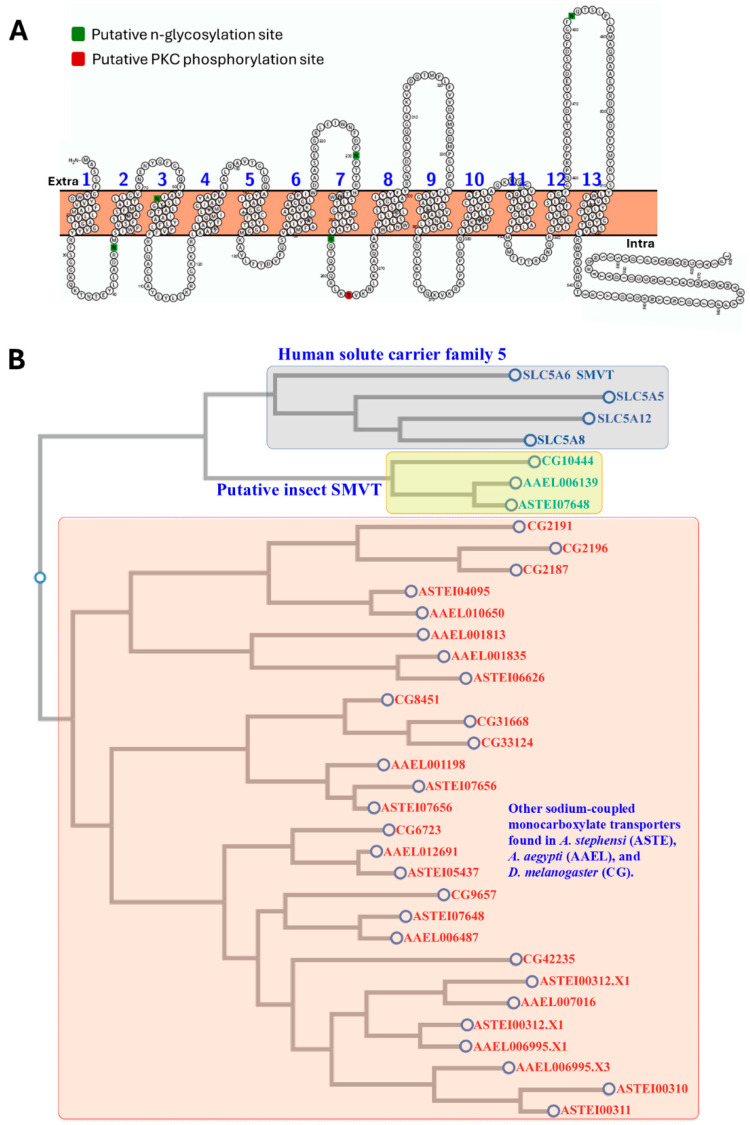
Predicted tertiary structure and phylogenetic tree of putative sodium-dependent multivitamin transporters (SMVTs). (**A**) Schematic representation of the predicted intracellular, extracellular, and transmembrane domains, as well as putative n-glycosylation and PKC phosphorylation sites in *A. stephensi* SMVT. Intracellular, extracellular, and alpha-helical transmembrane domains were detected by TMHMM 2.0 (https://services.healthtech.dtu.dk/services/TMHMM-2.0/, accessed on 12 October 2024). (**B**). A phylogenetic tree of human, mosquito, and fruit fly sodium-coupled monocarboxylate transporters. Multiple sequence alignment and phylogenetic tree construction were performed using CLUSTALW at GenomeNet (https://www.genome.jp/tools-bin/clustalw, accessed on 12 October 2024).

Using known amino acid sequences for the human SMVT and DmelSMVT proteins, we identified the putative *A. stephensi* SMVT ortholog, ASTEI07648, in the genome following a BLASTX search (NCBI). The predicted AsteSMVT protein is 60% similar and 41% identical to human SMVT and 77% similar and 59% identical to DmelSMVT. As with the human and fruit fly SMVTs, AsteSMVT includes 13 predicted transmembrane domains, with an intracellular C-terminus and extracellular N-terminus (Figure 5A). In humans, SMVT is regulated in part through a combination of n-glycosylation and protein kinase C (PKC) phosphorylation. AsteSMVT has four putative n-glycosylation sites (Asp-46, Asp-230, Asp-255, and Asp-482; green) [17]. Human SMVT has three PKC phosphorylation sites with one, Thr-265 (red), that is conserved in AsteSMVT. The conserved primary sequence, predicted secondary structure, and post-translational modification sites strongly suggest that ASTEI07648 encodes a putative AsteSMVT. ClustalW was used to construct a multiple sequence alignment and generate a phylogenetic tree (Figure 5B).

### 3.5. Tissue Distribution of AsteSMVT Transcript

**Figure 6:**
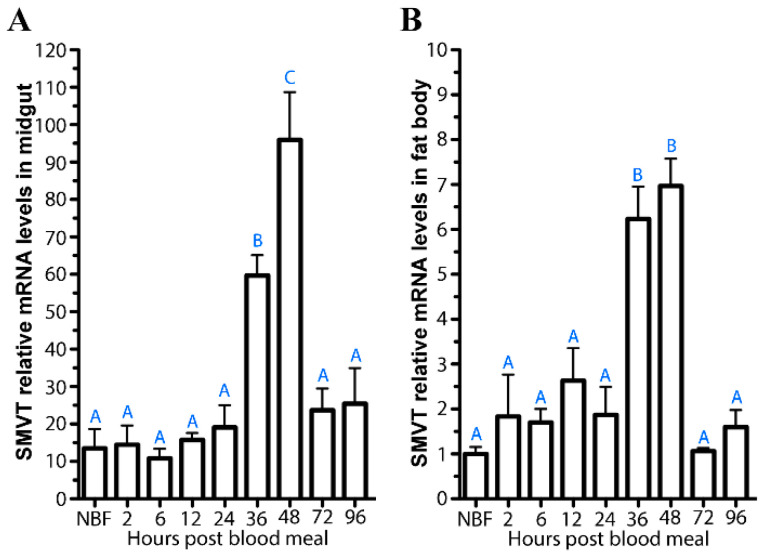
*AsteSMVT* transcript expression in the midgut (**A**) and fat body (**B**) throughout a reproductive cycle. qPCR was used to detect *AsteSMVT* transcript expression prior to blood feeding (NBF) and at various timepoints following a blood meal (2–96 h). Different letters indicate statistically significant differences (one-way ANOVA and Tukey’s post hoc).

We used qPCR to determine *AsteSMVT* transcript expression levels in the mosquito midgut and fat body (Figure 6A,B), as both tissues are key for Pan uptake, metabolism, and the host response to parasite infection. Ingested Pan must be mobilized across tissues, while Pan must be transported into the fat body for CoA synthesis, which plays a crucial role in the downstream processes of fatty acid synthesis and vitellogenesis. We examined *AsteSMVT* transcript expression NBF females and throughout the reproductive cycle (2–96 h PBM). In both the midgut and fat body, we observed dramatic increases in *AsteSMVT* transcript levels from 36–48 h PBM, timing that coincides with peak yolk protein synthesis during vitellogenesis. This pattern could reflect replenishment of AsteSMVT following resource intensive production of eggs.

### 3.6. Validation of AsteSMVT as a Pan and Biotin Transporter

**Figure 7:**
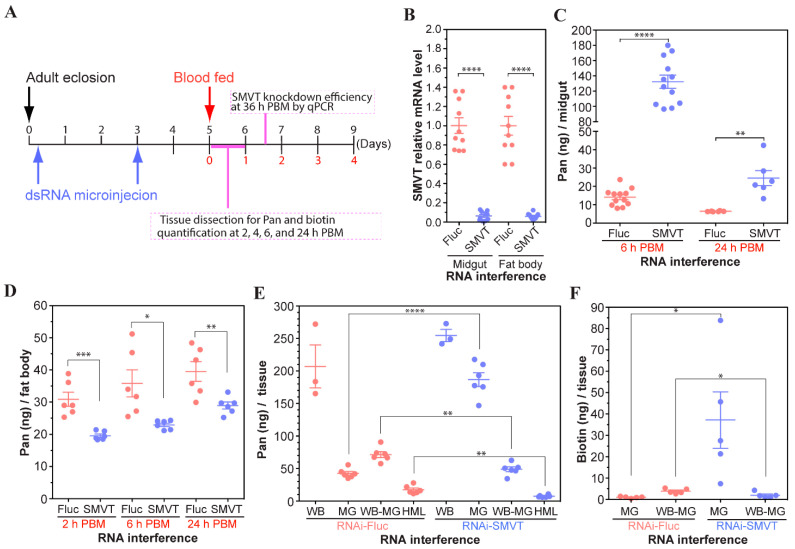
Impact of *AsteSMVT* RNAi knockdown on midgut and body Pan and biotin levels. (**A**). Schematic of RNAi knockdown protocol showing the timing of dsRNA microinjection, blood feeding, and tissue dissection for Pan and biotin determination. (**B**). Efficiency of *AsteSMVT* transcript knockdown as quantified by qPCR. (**C**). Pan levels were determined from dissected fat body at 2, 6, and 24 h PBM. Each dot represents fat bodies pooled from five RNAi treated mosquitoes (N = 30) and bars represent the mean and standard error. (**D**). Pan levels were determined from dissected midgut at 6 and 24 h PBM. Each dot contains midguts pooled from ten RNAi treated mosquitoes (N = 60–120). (**E**). Pan levels were determined from whole body (WB) and dissected midgut (MG), carcass (whole body minus midgut, WB–MG), and hemolymph (HML) at 4 h PBM (N = 15–30). (**F**). Biotin levels were determined using dissected midguts (MG) and carcasses (whole body minus midgut, WB–MG) at 4 h PBM (N = 25). Significant differences were determined using the unpaired Student’s *t*-test (* *p* < 0.05, ** *p* < 0.01, *** *p* < 0.001, and **** *p* < 0.0001).

To assess whether AsteSMVT functioned in Pan uptake and transport, we used RNAi to knock down transcript expression. Mosquitoes were injected with *dsAsteSMVT* or *dsFluc* (firefly luciferase, negative control) 4 and 72 h after adult emergence and allowed to recover until 92 h post-emergence (Figure 7A). Recovered mosquitoes were provided with a blood meal and knockdown efficiency was assessed at 48 h PBM in the midgut (96% knockdown) and fat body (91% knockdown; Figure 7B). In the midgut, *AsteSMVT* knockdown led to a 10-fold increase in Pan levels at 6 h and a 4-fold increase by 24 h PBM relative to *dsFluc*-injected controls (Figure 7C). These data suggest that loss of AsteSMVT led to Pan retention in the midgut, which could be eliminated in the feces. In the fat body, knockdown of *AsteSMVT* led to a significant decrease in Pan levels in this tissue in response to a Pan-supplemented blood meal (Figure 7D). In fact, there was a nearly 50% reduction in Pan uptake by the fat body relative to *dsFluc* injected and non-injected controls. This could result from reduced Pan uptake from the midgut lumen by midgut AsteSMVT, reduced uptake into the fat body by fat body AsteSMVT, or a combination thereof. Finally, we observed a significant decrease of Pan in both the carcass (minus midgut) and hemolymph at 4 h PBM (Figure 7E). Additional studies are needed to elucidate tissue-specific roles of AsteSMVT.

As described above, SMVTs in humans can transport both Pan and biotin. To assess whether the putative AsteSMVT could also transport biotin, we assessed biotin levels in midguts and whole bodies (minus midguts) of female *A. stephensi* injected with *dsAsteSMVT* or *dsFluc* (control) and provisioned with a blood meal supplemented with 100 ng/µL biotin. Biotin levels were assessed using the standard assay for foodstuffs from AOAC International (Cunniff, 1995), with bacteria grown in biotin-deficient Difco medium given that biotin, like Pan, is essential for *Lactobacillus plantarum* growth. This protocol is detailed in Section 2.2. As with Pan, knockdown of *AsteSMVT* led to retention of biotin in the midgut relative to *dsFluc* controls (Figure 7F), likely due to reduced biotin uptake by AsteSMVT from the blood meal. Biotin levels in the body minus midgut (WB-MG) were also reduced by ~50% in *AsteSMVT* knockdown mosquitoes relative to *dsFluc* controls, suggesting that AsteSMVT is also responsible for the uptake and movement of biotin in the mosquito.

### 3.7. Impact of Pan Supplementation and AsteSMVT on A. stephensi Reproduction and Survival

**Figure 8:**
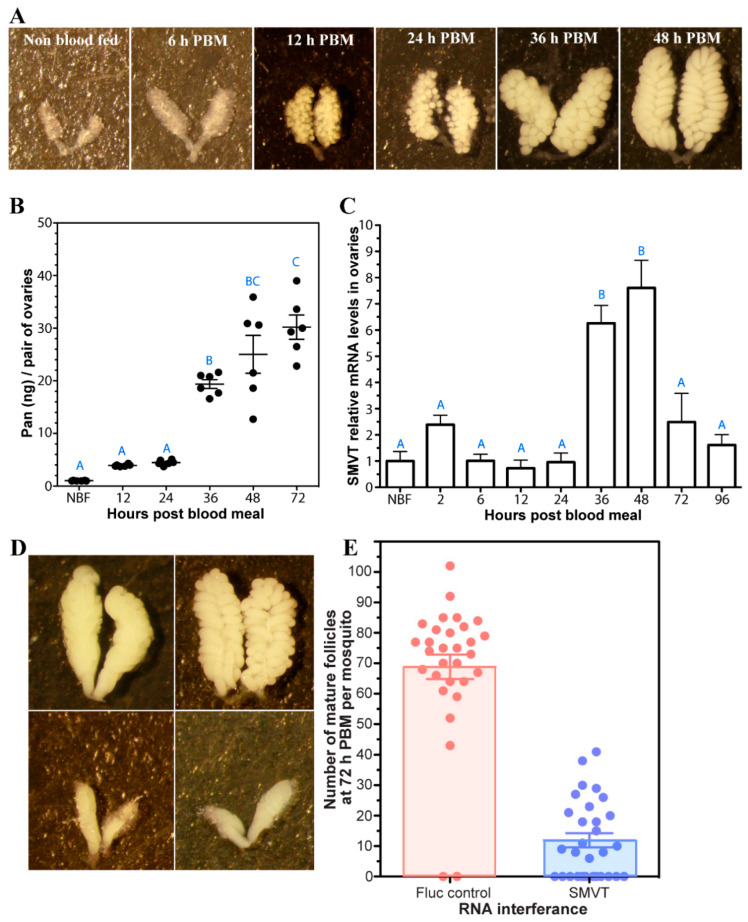
Pan and *AsteSMVT* transcript levels in *A. stephensi* ovaries during follicle development. (**A**). Ovaries prior to blood feeding (NBF) and at 6, 12, 24, 36, and 48 h PBM. All ovaries were imaged at an identical magnification (20×) (**B**). Pan levels were measured by *Lactobacillus plantarum* microbiological assay in ovaries dissected from NBF mosquitoes and mosquitoes at 12, 24, 36, and 48 h PBM. Each dot represents 10 pairs of ovaries and bars represent the mean and standard error. Different letters indicate significant differences (one-way ANOVA, N = 6). (**C**). Relative *AsteSMVT* mRNA expression in ovaries during development. qPCR was performed on three separate cohorts of mosquitoes using *AsteSMVT* gene-specific primers and normalized to ribosomal protein S7 (*AsteRPS7*). Both *AsteSMVT* and *AsteRPS7* showed a single peak in their respective melting curves. Relative levels were calculated by normalizing to NBF. Different letters indicate significantly different expression levels (*p* < 0.05). (**D**). Knockdown of *AsteSMVT* led to a significant decrease in the number of fully developed follicles at 72 h PBM. Upper photos are representative ovaries from controls injected with *dsFluc* and bottom photos are representative of ovaries from *AsteSMVT* knockdown mosquitoes. All ovaries were imaged at an identical magnification (20×) (**E**). Number of developed follicles at 72 h in females injected with *dsFluc* or *dsAsteSMVT* (*n* = 30; *p* < 0.001).

During the first 48 h PBM, the female mosquito reproductive cycle is coordinated by hormonal cues that lead to the synchronous development of nearly 200 ovarian follicles (Figure 8A) with provisioning of yolk proteins, lipids, carbohydrates, and other metabolites. Given that maternal CoA precursors are provisioned to developing eggs in *Drosophila* [18], we asked whether Pan was similarly required for *A. stephensi* egg development. We observed that ovary Pan levels increased significantly during the nutrient uptake stage of egg development from 24 to 48 h PBM (Figure 8B). In fact, Pan levels increased more than six-fold during the course of vitellogenesis, when the developing follicles are provisioned with nutrients. Interestingly, along with increased ovarian Pan levels, we observed a corresponding increase in *AsteSMVT* expression during this same period, suggesting that the ovaries were increasing their capacity to take up Pan (Figure 8C). We also observed that knockdown of *AsteSMVT* led to a significant reduction in the number of developing follicles (Figure 8D), likely due to reduced Pan import into the developing follicles.

**Figure 9:**
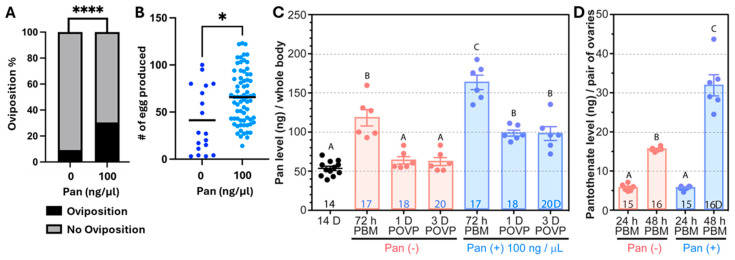
Impact of Pan supplementation on reproduction in 14 d old *A. stephensi*. (**A**). Percentage of 14 d old mosquitoes ovipositing when the blood meal was supplemented with 100 ng/µL Pan or without Pan (*n* = 195 control females, *n* = 261 Pan-treated females from four biological replicates; **** *p* < 0.0001). (**B**). Number of eggs laid per ovipositing female (*n* = 18 control females, *n* = 70 Pan-treated females from four biological replicates; * *p* < 0.05). Pan levels in the whole bodies (**C**) and ovaries (**D**) of 14 d old female mosquitoes provisioned with Pan. Each dot represents a pool of 5 mosquitoes and bars represent the mean and standard error. The age of mosquitoes or time post-oviposition (POVP) when the sample was collected is indicated at the bottom of each bar. Different letters indicate statistically significant differences (one-way ANOVA and Tukey’s post hoc).

Increased ovarian Pan coupled with increased *AsteSMVT* expression suggested that Pan may be essential for egg development. Further, Pan levels in female *A. stephensi* decline with increasing age, consistent with declining reproductive output. Accordingly, we examined the impact of Pan supplementation on 14 d old female *A. stephensi*, when Pan levels were at their lowest (Figure 1H). At this age, fewer than 10% of blood fed controls produced any eggs, compared with nearly 30% of females supplemented with Pan in the blood meal (Figure 9A). Of mosquitoes that laid eggs, significantly more eggs were produced by females provisioned with Pan in the blood meal (Figure 9B), with nearly half of the control mosquitoes producing clutches with fewer than 30 eggs. Pan supplementation of 14 d old mosquitoes significantly increased whole body Pan levels prior to oviposition (72 h PBM) and at 1 and 3 d after oviposition (Figure 7C). In contrast to an observed 6-fold increase in ovarian Pan levels in unsupplemented young females between 24 h and 48 h PBM (Figure 8B), the increase in ovarian Pan levels in the same time period in unsupplemented 14 d old females was less than 3-fold (Figure 9D). Supplementation of 14 d old females with Pan, however, recovered this fold increase to that observed in young females (Figure 7D), suggesting that supplementation-enhanced ovarian Pan directly contributed to increased oviposition and clutch sizes (Figure 7D).

**Figure 10:**
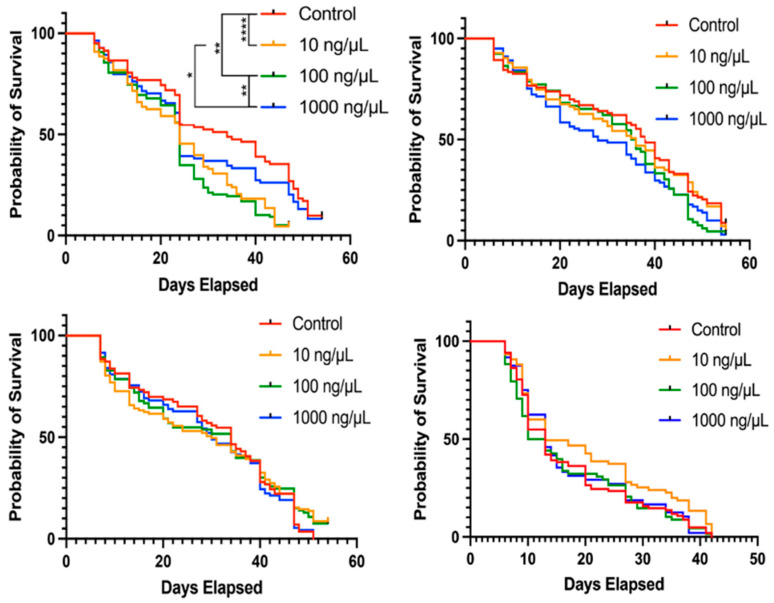
Pan supplementation via blood meal did not impact adult female *A. stephensi* survival. Survival curves from four separate cohorts showing the daily probability of survival for adult females supplemented with 0, 10, 100 or 1000 ng/µL Pan via blood meal. Dead mosquitoes were counted and removed daily (*n* = 1443 females across all replicates, *n* ≈ 100 females per treatment per replicate); * *p* < 0.05, ** *p* < 0.01, **** *p* < 0.0001). Kaplan–Meier survival analysis was used to test the variation in survival distribution of mosquitoes in each cohort while Log-rank test was used to compare each treatment group with the control.

We also asked whether Pan supplementation impacted adult female survival. Female mosquitoes were provisioned with 0 to 1000 ng/µL weekly via blood meal. Adult survivorship was measured daily until the final mosquito perished. Over three biological replicates, we observed a slight trend towards reduction in adult lifespan in the Pan treated groups, although only the first replicate showed significant differences in lifespans (Figure 10). Thus, provisioning of additional Pan does not appear to have a major impact on adult female survival, perhaps reflecting the substantial investments of Pan in reproduction during later gonotrophic cycles.

**Figure 11:**
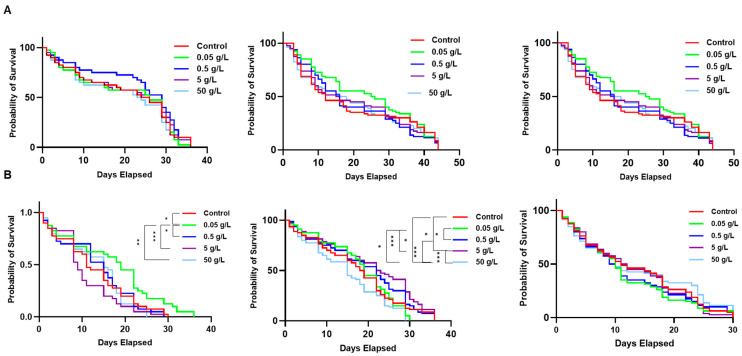
Pan supplementation via water-soaked cotton pads did not impact adult *A. stephensi* survival. Survival curves showing the daily probability of survival for females (**A**) and males (**B**) supplemented with 0, 0.05, 0.5, 5 or 50 g/L Pan via water-soaked cotton balls. Cotton balls were replaced daily, and dead mosquitoes were counted and removed daily (*n* = 40 males and females per group in replicate 1, *n* = 80 males and females per group in replicates 2 and 3; * *p* < 0.05, ** *p* < 0.01, *** *p* < 0.001). Kaplan–Meier survival analysis was used to test the variation in survival distribution of mosquitoes in each cohort while Log-rank test was used to compare each treatment group with the control.

Similar survival results were obtained when Pan was supplemented via water-soaked cotton pads, provided with a sugar cube for carbohydrates, instead of a blood meal. Provisioning of Pan-supplemented water to female mosquitoes resulted in a modest reduction in female survival in one biological replicate and no significant difference in the other two replicates (Figure 11A). Likewise, in male mosquitoes provisioned with Pan via sugar feeding, in one replicate we observed no effects on lifespan and in the other two we observed mixed results between Pan concentrations and replicates (Figure 11B). Taken together, Pan supplementation in water had no consistent impacts on adult female and male *A. stephensi* survival.

## 4. Discussion

Pan is the precursor for CoA and acetyl-CoA, which are essential for numerous metabolic pathways in eukaryotic organisms [19]. In the malaria parasite life cycle, these metabolites are essential not only for the vertebrate and mosquito hosts, but also for parasite growth and development [20]. Despite this, few to no studies have been published on Pan levels in mosquitoes, Pan storage and metabolism in mosquitoes, transfer among tissues across lifespan, and in reproduction. In this study, we explored Pan levels in *A. stephensi* life stages and tissues, how Pan is acquired, how much is used, and the fate of excess Pan. In this context, we identified a putative Pan/biotin transporter and confirmed its role in transporting Pan and biotin to *A. stephensi* tissues. Finally, we analyzed the impact of Pan on mosquito lifespan and reproduction, two physiological processes that are also key for *Plasmodium* parasite transmission [21]. Taken together, this study provides substantial support for our focus on a strategy to disrupt malaria parasite development and transmission by limiting Pan reserves in the mosquito vector [4,5,7].

Our data indicate that Pan is acquired during the immature stages, with the highest levels of Pan in laboratory-reared *A. stephensi* observed after adult eclosion (Figure 1H). This is to be expected since Pan is exclusively synthesized by plants and bacteria, which constitute much of the larval mosquito diet [22]. Both blood and nectar, the two food sources for adult mosquitoes, also contain Pan, which can contribute to acquisition of this vitamin during the adult stage [7]. In the absence of supplementation, Pan levels decline, suggesting that midgut microbiota under laboratory conditions do not synthesize sufficient Pan to meet adult female metabolic requirements. Specifically, Pan levels increased following an unsupplemented blood meal, then declined over the next several days (Figure 4D). In females, Pan levels were correlated with body mass, with larger females having correspondingly greater levels of Pan. Interestingly, body size was not correlated with Pan levels in male mosquitoes, suggesting that their shorter lifespan and lower reproductive investment may be less dependent on Pan bioavailability.

In adult female *A. stephensi*, our data confirmed that Pan supplementation increased whole body Pan levels, with excretion of excess Pan. Notably, even a single day of access to Pan supplemented 10% sucrose was associated with increased Pan levels being sustained through three weeks, possibly due to the measured release of the crop contents into the midgut over approximately 2 d, which may allow for greater uptake of Pan through the midgut (Figure 3). In contrast, Pan supplementation in the blood meal was associated with increased Pan levels at 4 d after the blood meal, but this increase was not evident at 10 d PBM (Figure 4D). This could be due in part to Pan provisioning to the developing oocytes as discussed below. Despite this acquisition and use, excess supplemented Pan is lost during the first hour of blood feeding when diuresis eliminates water from the blood meal. Accordingly, there is a limit to how much Pan can be transported from the midgut into the mosquito body, suggesting a critical role for Pan transport. The distribution of Pan in the mosquito also changes during reproductive cycles, with the most dramatic changes occurring in the ovaries and thorax. In the ovaries, we observed a consistent increase in Pan throughout the reproductive cycle, indicating that this vitamin is essential for egg development. In the thorax, we observed a dramatic decrease in Pan levels throughout the first reproductive cycle and consistent low Pan levels throughout the second, indicating that Pan may be mobilized from thoracic tissue to provision the developing clutch of eggs. The thorax consists primarily of muscle tissue, although epithelial, nervous, and other tissues are present as well. Thus, it will be interesting in future studies to explore whether Pan depletion in the thoracic flight muscles leads to decreased adult female activity and whether Pan supplementation reverses these effects.

As discussed above, SMVTs in humans and fruit flies have been shown to transport Pan, biotin, lipoic acid, and iodide, with an obligate role only for Pan transport. *AsteSMVT* is broadly expressed in *A. stephensi* females, including the midgut, fat body, and ovaries. In fact, we observed significantly increased *AsteSMVT* expression in all three tissues late in the vitellogenic cycle (36–48 h PBM; Figure 6 and Figure 8C). RNAi supported our conclusion that AsteSMVT is responsible for the transport of both Pan and biotin in female *A. stephensi*. In females with reduced *AsteSMVT* expression, we observed a significant increase in midgut Pan and reduced levels in the carcass, fat body, and hemolymph. This suggests that Pan was retained in the midgut lumen versus transported across the midgut epithelium and into the hemolymph. An associated reduction in fat body Pan suggests that knockdown here additively reduces transport into this tissue. Biotin levels were similarly affected, with retention of this vitamin in the midgut lumen.

In previous work, we demonstrated that reduced Pan levels in female mosquitoes can limit *Plasmodium* development [4]. Further, we showed that increasing the conversion of Pan to CoA using small molecule activators of PanK, called pantazines, does not impact mosquito fitness since CoA is still available to the mosquito [5]. However, a decline in Pan, as occurs with increasing female age or via knockdown of *AsteSMVT* expression, is associated with reduced reproductive output. These observations affirm that Pan is provisioned into the ovaries during a reproductive cycle, with Pan levels in the developing oocytes increasing dramatically from 36–72 h after the blood meal. This time period corresponds with the rapid provisioning of the oocyte with yolk proteins, lipids, and other nutrients. Expression of *AsteSMVT* is also increased in the ovaries during this time, suggesting that Pan uptake is critical for egg development. *AsteSMVT* RNAi was associated with a dramatic reduction in follicle development when Pan transport to the ovaries was reduced (Figure 8), suggesting that some level of Pan is essential for adult female *A. stephensi* to produce a full clutch of eggs.

As *Anopheles* female mosquitoes age, their reproductive output is reduced following each successive reproductive cycle [23]. Interestingly, this decrease in fecundity corresponds with our above observations that Pan also decreases as mosquitoes age. These data, coupled with our observations that Pan is essential for follicle development, led us to assess whether Pan supplementation could increase egg production in older mosquitoes. When Pan was provisioned to 14 d old females via a blood meal, we observed an increase in both the percentage of mosquitoes that laid eggs and the average clutch size of those mosquitoes that produced offspring. Provisioning Pan to 14 d female mosquitoes resulted in whole body Pan levels consistent with younger mosquitoes (~100–150 ng; Figure 1H and Figure 9C), which led to increased ovarian Pan level, also consistent with younger mosquitoes (~30 ng/ovary pair; Figure 8B and Figure 9D). Thus, reduced Pan bioavailability is at least partially responsible for reduced egg production in older mosquitoes. Interestingly, we observed significantly increased Pan levels in the ovaries and whole bodies of unsupplemented females at 48 and 72 h PBM, and the source of this Pan is unclear. Some Pan may have been acquired through the blood meal, but Pan can also be scavenged through CoA degradation, which may be more active as Pan levels decrease in older mosquitoes. Pan acquisition, especially as mosquitoes age, will be an interesting topic for future exploration, especially as it influences mosquito reproduction, survival, metabolism, and immunity.

## 5. Conclusions

This work expands our understanding of Pan-dependent biology in *Anopheles* mosquitoes and supports the feasibility of manipulating CoA biosynthesis to limit *Plasmodium* development in the mosquito host [4,5]. The putative Pan transporter in *A. stephensi* is another potential target for modulating mosquito Pan levels and limiting Pan access by the malaria parasite. Finally, our discovery that Pan is essential for mosquito reproduction also suggests a potential multi-hit strategy to couple targeted inhibition of malaria parasite development and reproduction in *A. stephensi*.

## Data Availability

The data presented in this study are available on request from the corresponding author.

## Supplementary Materials

The following supporting information can be downloaded at: https://www.mdpi.com/article/10.3390/biom15010059/s1, Table S1. Primers used in these studies.

## References

[1] WHO (2023). World Malaria Report 2023.

[2] Al-Eryani S.M., Irish S.R., Carter T.E., Lenhart A., Aljasari A., Montoya L.F., Awash A.A., Mohammed E., Ali S., Esmail M.A. (2023). Public health impact of the spread of *Anopheles stephensi* in the WHO Eastern Mediterranean Region countries in Horn of Africa and Yemen: Need for integrated vector surveillance and control. Malar. J..

[3] Divo A.A., Geary T.G., Davis N.L., Jensen J.B. (1985). Nutritional requirements of *Plasmodium falciparum* in culture. I. Exogenously supplied dialyzable components necessary for continuous growth. J. Protozool..

[4] Simão-Gurge R.M., Thakre N., Strickland J., Isoe J., Delacruz L.R., Torrevillas B.K., Rodriguez A.M., Riehle M.A., Luckhart S. (2021). Activation of *Anopheles stephensi* pantothenate kinase and coenzyme a biosynthesis reduces infection with diverse *Plasmodium* species in the mosquito host. Biomolecules.

[5] Thakre N., Gurge R.M.S., Isoe J., Kivi H., Strickland J., Delacruz L.R., Rodriguez A.M., Haney R., Sadeghi R., Joy T. (2022). Manipulation of pantothenate kinase in *Anopheles stephensi* suppresses pantothenate levels with minimal impacts on mosquito fitness. Insect Biochem. Mol. Biol..

[6] Rock C.O., Calder R.B., Karim M.A., Jackowski S. (2000). Pantothenate kinase regulation of the intracellular concentration of coenzyme A. J. Biol. Chem..

[7] Riske B.F., Luckhart S., Riehle M.A. (2023). Starving the beast: Limiting coenzyme A biosynthesis to prevent disease and transmission in malaria. Int. J. Mol. Sci..

[8] Dutt Vadlapudi A., Krishna Vadlapatla R., K Mitra A. (2012). Sodium dependent multivitamin transporter (SMVT): A potential target for drug delivery. Curr. Drug Targets.

[9] Gyimesi G., Pujol-Giménez J., Kanai Y., Hediger M.A. (2020). Sodium-coupled glucose transport, the SLC5 family, and therapeutically relevant inhibitors: From molecular discovery to clinical application. Pflüg. Arch. Eur. J. Physiol..

[10] Quick M., Shi L. (2015). The sodium/multivitamin transporter: A multipotent system with therapeutic implications. Vitam. Horm..

[11] Daberkow R.L., White B.R., Cederberg R.A., Griffin J.B., Zempleni J. (2003). Monocarboxylate transporter 1 mediates biotin uptake in human peripheral blood mononuclear cells. J. Nutr..

[12] Dutta D., Dobson A.J., Houtz P.L., Gläßer C., Revah J., Korzelius J., Patel P.H., Edgar B.A., Buchon N. (2015). Regional cell-specific transcriptome mapping reveals regulatory complexity in the adult *Drosophila* midgut. Cell Rep..

[13] Neophytou C., Pitsouli C. (2022). Biotin controls intestinal stem cell mitosis and host-microbiome interactions. Cell Rep..

[14] Cunniff P., Washington D. (1997). Official methods of analysis of AOAC International. J. AOAC Int.

[15] Nasci R.S. (1986). The size of emerging and host-seeking *Aedes aegypti* and the relation of size to blood-feeding success in the field. J. Am. Mosq. Control Assoc..

[16] Graumans W., Heutink R., van Gemert G.-J., van de Vegte-Bolmer M., Bousema T., Collins K.A. (2020). A mosquito feeding assay to examine *Plasmodium* transmission to mosquitoes using small blood volumes in 3D printed nano-feeders. Parasit. Vectors.

[17] Ghosal A., Subramanian V.S., Said H.M. (2011). Role of the putative N-glycosylation and PKC-phosphorylation sites of the human sodium-dependent multivitamin transporter (hSMVT) in function and regulation. Biochim. Biophys. Acta. Biomembr..

[18] Yu Y., van der Zwaag M., Wedman J.J., Permentier H., Plomp N., Jia X., Kanon B., Eggens-Meijer E., Buist G., Harmsen H. (2022). Coenzyme A precursors flow from mother to zygote and from microbiome to host. Mol. Cell.

[19] Depeint F., Bruce W.R., Shangari N., Mehta R., O’Brien P.J. (2006). Mitochondrial function and toxicity: Role of the B vitamin family on mitochondrial energy metabolism. Chem. Biol. Interact..

[20] Tjhin E.T., Spry C., Sewell A.L., Hoegl A., Barnard L., Sexton A.E., Siddiqui G., Howieson V.M., Maier A.G., Creek D.J. (2018). Mutations in the pantothenate kinase of *Plasmodium falciparum* confer diverse sensitivity profiles to antiplasmodial pantothenate analogues. PLoS Pathog..

[21] Brady O.J., Godfray H.C.J., Tatem A.J., Gething P.W., Cohen J.M., McKenzie F.E., Perkins T.A., Reiner R.C., Tusting L.S., Sinka M.E. (2016). Vectorial capacity and vector control: Reconsidering sensitivity to parameters for malaria elimination. Trans. R. Soc. Trop. Med. Hyg..

[22] Coxon K., Chakauya E., Ottenhof H., Whitney H., Blundell T., Abell C., Smith A. (2005). Pantothenate biosynthesis in higher plants. Biochem. Soc. Trans..

[23] Fernandes L., Briegel H. (2005). Reproductive physiology of *Anopheles gambiae* and *Anopheles atroparvus*. J. Vector Ecol..

