# Supplementary material for: Characterization of the Sodium Multi-Vitamin Transporter in the Mosquito Anopheles stephensi and Its Capacity to Mobilize Pantothenate and Biotin
Source: Biomolecules. 2025 Jan 3;15(1):59. doi: 10.3390/biom15010059 (PMC11764013; doi:10.3390/biom15010059)
Supplement: Supplementary file 1 [file biomolecules-15-00059-s001.zip › biomolecules-3313370-supplementary.pdf]

Supplemental Table S1. Gene-specific primers used for RNAi and qPCR.

Genes			PCR size
Gene-specific primers used for RNAi			
Sodium-dependent multivitamin transporter	Forward	TGGCGTCATCGTTCGGTGT	515 bp
	Reverse	GTAGAACGTGCAGATGCC	
Firefly Luciferase, Fluc	Forward	AGCACTCTGATTGACAAATACGA	548 bp
pGL3-Basic Vector	Reverse	AGTTCACCGGCGTCATCGTC	
Gene-specific primers used for qPCR			
Sodium-dependent multivitamin transporter	Forward	GACCAGGCGTACTCGATCCT	119 bp
	Reverse	ATGGCGGCAAACATAAGCAC	
Ribosomal protein S7	Forward	CGTGAGGTCGAGTTCAACAA	107 bp
	Reverse	TTCTCCAATTCACGCACCAG	

T7 promoter sequence (5' TAATACGACTCACTATAGGGAGA 3') was added in 5' of each RNAi primer.
